# Mitochondrial metabolism and epigenetic crosstalk drive the SASP

**DOI:** 10.21203/rs.3.rs-5278203/v1

**Published:** 2024-12-05

**Authors:** Helene Martini, Jodie Birch, Francisco Marques, Stella Victorelli, Anthony Lagnado, Nicholas Pirius, Ana Franco, Gung Lee, Yeaeun Han, Jennifer Rowsey, Alexandre Gaspar-Maia, Aaron Havas, Rabi Murad, Xue Lei, Rebecca Porritt, Oliver Maddocks, Diana Jurk, Sundeep Khosla, Peter Adams, Joao Passos

**Affiliations:** Mayo Clinic; MRC London Institute of Medical Sciences; Albert Einstein College of Medicine; Mayo Clinic; Mayo Clinic; Mayo Clinic; Mayo Clinic; Mayo Clinic; Mayo Clinic; Mayo Clinic; Mayo Clinic; Sanford Burnham Prebys Medical Discovery Institute; University of California, Irvine; Sanford Burnham Prebys MDI; Sanford Burnham Prebys Medical Discovery Institute; Faeth Therapeutics; Mayo Clinic; Mayo Clinic; Sanford Burnham Prebys Medical Discovery Institute; Mayo Clinic

## Abstract

Senescent cells drive tissue dysfunction through the senescence-associated secretory phenotype (SASP). We uncovered a central role for mitochondria in the epigenetic regulation of the SASP, where mitochondrial-derived metabolites, specifically citrate and acetyl-CoA, fuel histone acetylation at SASP gene loci, promoting their expression. We identified the mitochondrial citrate carrier (SLC25A1) and ATP-citrate lyase (ACLY) as critical for this process. Inhibiting these pathways selectively suppresses SASP without affecting cell cycle arrest, highlighting their potential as therapeutic targets for age-related inflammation. Notably, SLC25A1 inhibition reduces systemic inflammation and extends healthspan in aged mice, establishing mitochondrial metabolism as pivotal to the epigenetic control of aging.

## Introduction

Cellular senescence is a key response to various cellular stresses, characterized by a permanent cell-cycle arrest and the secretion of a pro-inflammatory cocktail of factors known as the Senescence-Associated Secretory Phenotype (SASP). Senescence serves important roles in embryonic development^1,2^, tumor suppression^3^ and tissue repair^4,5^. However, the prolonged activation of the SASP can lead to chronic inflammation, driving tissue degeneration, aging, and diseases, including cancer. This dual nature makes the SASP a focal point for understanding both the protective and detrimental effects of senescence.

Mitochondria, central to cellular metabolism, undergo significant changes during senescence^6,7^. These include altered morphology, impaired dynamics, and metabolic reprogramming, particularly involving the tricarboxylic acid (TCA) cycle^8^. Elevated levels of TCA cycle intermediates, such as acetyl-CoA, fumarate, and succinate, accumulate in senescent cells.^8^ These metabolites not only fuel biosynthetic and energy-producing pathways but also influence cellular signaling and epigenetic regulation. For instance, succinate and fumarate are recognized as oncometabolites due to their roles in promoting tumorigenesis^9^, and acetyl-CoA is crucial for histone acetylation, a key epigenetic modification that governs gene expression^10^.

Despite the well-established role of mitochondria in energy production and redox control, their broader contribution to regulating the SASP through epigenetic modifications remains underexplored. Our group has shown that mitochondria are central to SASP regulation^11^, suggesting that mitochondrial metabolites may serve as key regulators of epigenetic changes that drive SASP gene expression. Specifically, we hypothesize that mitochondrial-derived metabolites, such as citrate and acetyl-CoA, mediate epigenetic modifications that promote SASP components.

In this study, we sought to elucidate the mechanisms by which mitochondrial metabolic remodeling contributes to the regulation of the SASP through epigenetic pathways. Our results demonstrate that mitochondrial function is critical for H3K27 acetylation at SASP gene loci. Specifically, we identified that the inhibition of pathways responsible for mitochondrial citrate production, its export, and its subsequent conversion to acetyl-CoA in the cytoplasm/nucleus are central to the regulation of the SASP. Importantly, we found that inhibition of this process in vivo decreases inflammatory markers and improves healthspan in aged mice.

## Results

### Mitochondria influence histone acetylation at SASP genes.

Mitochondria, through their metabolic activities, produce and regulate various metabolites that influence multiple cellular functions, including the epigenome. Notably, acetyl-CoA acts as the exclusive donor for protein acetylation, such as histone acetylation^12,13^. This process is linked to enhanced gene expression by loosening chromatin structure, which increases accessibility for transcription machinery.

**Figure 1:**
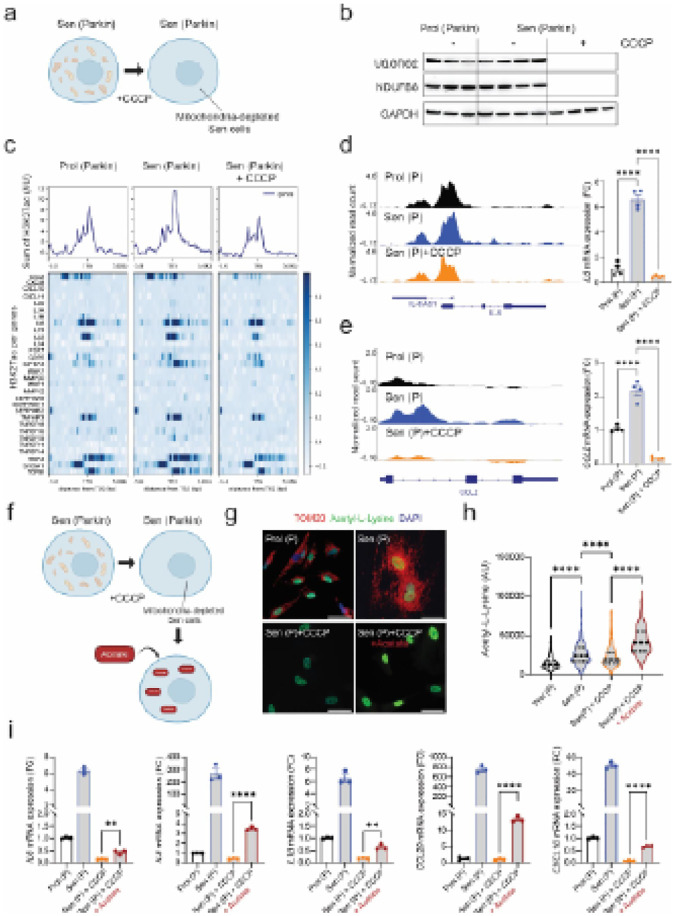
Mitochondria modulate histone acetylation at SASP loci. **(a)** Schematic representation of the experimental setup. **(b)** Western blot analysis of mitochondrial proteins UQCRC2 and NDUFB8, confirming the complete loss of mitochondria in IMR90 Parkin senescent cells (Sen) following CCCP treatment**. c**. Metaplot and heatmap analysis of SASP genes showing decreased histone acetylation (H3K27ac) in senescent cells lacking mitochondria (Sen+CCCP). Top: Metaplot depicting a composite sum of all normalized H3K27ac enrichment at SASP gene signals; Bottom: heatmap of H3K27ac enrichment normalized by total H3 (±5kb from TSS). Data are averaged from n=3 independent experiments. **(d-e).** Browser track (left panel) and mRNA expression (right panel) of IL6 **(d)** and CCL2 **(e)** genes displaying H3K27ac distribution. qPCR data, n=4. **(f).** Scheme of the experimental workflow**. (g)**. Representative immunofluorescence images of nuclear acetylated lysine (green) and mitochondria staining (TOMM20, red). Scale bar 50μm. **(h)**. Quanti cation of nuclear acetyllysine per cell from n=3 independent experiments. **(i).** mRNA expression levels of various SASP genes (n=3 per condition). Data are expressed as Mean ± S.E.M. One way ANOVA or Student’s t-test *p<0.05; **p<0.01; ***p<0.001; ****p<0.0001.

To investigate the role of mitochondria in regulating histone acetylation during senescence, we employed a system we previously developed to generate cells lacking mitochondria. Human fibroblasts were stably transduced with Parkin and treated with CCCP, inducing widespread mitophagy and creating cells completely depleted of mitochondria (Fig. 1a). In this model, we analyzed a list of 53 senescence-associated secretory phenotype (SASP) genes, identified by RNA sequencing, that were significantly upregulated in senescent cells and downregulated following mitochondrial clearance (Extended data Fig.1a). To explore the chromatin landscape of these genes, we examined nine published chromatin immunoprecipitation sequencing (ChIP-seq) datasets from various senescence models, including senescence induced by radiation (Sen (IR)), etoposide (Sen (Etop)), oncogene (Sen (OIS) and replicative exhaustion (Sen (RS)). Across these datasets, we found that many of the SASP genes showed enrichment for different acetylated histone marks including H3K18ac, H4K5ac, H3122ac, H3K16ac, H3K23ac and H3K27ac^14–17^. However, the composition of acetylated SASP genes varied according to the inducing stimuli and/or histone mark (Extended Data Fig. 1b-l).

Next, we generated senescent human fibroblasts either with or without mitochondria and performed ChIP-seq using an antibody specific to acetylated histone H3 lysine 27 (H3K27ac). We confirmed that mitochondrial proteins were absent in mitochondria-depleted senescent cells (Fig. 1b). Analysis of SASP genes revealed a global enrichment of H3K27ac in senescent cells, which diminished following mitochondrial clearance (Fig. 1c). Representative examples of two SASP components, *IL6* and *CCL2,* along with corresponding mRNA expression levels, are shown in Fig. 1d–e.

This proof-of-concept experiment led us to hypothesize that mitochondria serve as a primary contributor to histone acetylation at SASP genes, thereby regulating their expression.

### Acetate induces histone acetylation and SASP.

While cells are impermeable to acetyl-CoA, exogenous acetate can be converted into acetyl-CoA by acetate-dependent acetyl-CoA synthetase 2 (ACSS2). To further investigate the role of acetyl-CoA in histone acetylation at SASP genes, we supplemented human fibroblasts with acetate (Extended Data Fig. 2a). Acetate supplementation resulted in increased global nuclear acetylation, as shown by enhanced pan-acetyl-L-lysine staining in the nuclei (Extended Data Fig. 2b) and elevated H3K27ac levels relative to total H3 (Extended Data Fig. 2c-d). Supporting our hypothesis that SASP gene expression can be driven by intracellular acetyl-CoA levels, acetate supplementation led to increased expression of SASP factors, including *IL6, IL8, IL1-β*, and *CCL2* (Extended Data Fig. 2e), but not cyclin-dependent kinase inhibitors p16 and p21 (Extended Data Fig.2f).

We next tested whether acetate supplementation could restore SASP expression in senescent cells lacking mitochondria, generated as before by Parkin-mediated mitophagy (Fig.1f). Our results showed that acetate supplementation increased nuclear pan-acetyl-lysine levels (Fig.1g and h) and partially restored the expression of several SASP components in senescent cells lacking mitochondria (Fig. 1i). These findings suggest that mitochondrial-dependent histone acetylation is a key limiting factor in regulating SASP gene expression.

### Upregulation of Mitochondrial pyruvate carrier (MPC) in senescence modulate the SASP.

**Figure 2:**
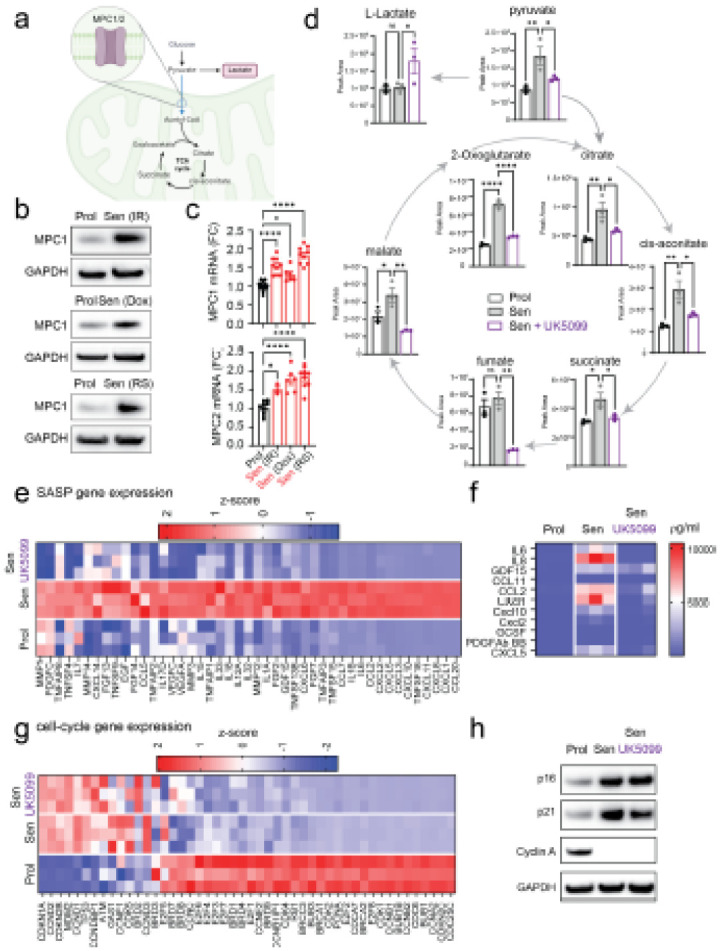
Upregulation of mitochondrial pyruvate carriers (MPC) in senescence modulates the SASP. **(a)** Schematic illustration of MPC localization and function within the mitochondria. **(b)** Western blot analysis showing increased MPC1 protein levels in different models of senescence: irradiation-induced (Sen(IR)), doxorubicin-induced (Sen(Dox)), and replicative senescence (Sen(RS)). **(c)** mRNA expression levels of *MPC1* and *MPC2* across senescence models. **(d)** Mass spectrometry quantification of TCA cycle metabolites in senescent cells following treatment with UK5099 (MPC inhibitor) (n=3 independent experiments). **(e)** Column-clustered heatmap showing differential expression of SASP genes in senescent cells, with downregulation following UK5099 treatment. Color intensity represents column Zscores (red: high expression, blue: low expression). **(f)** Cytokine array heatmap of 24-hour conditioned media from senescent cells, showing cytokine expression changes. **(g)** Column-clustered heatmap of cell cycle-associated genes in senescent cells, which are not affected by UK5099 treatment (color intensity as in panel e). **(h)** Western blot indicating that UK5099 does not reduce expression of p16 and p21, nor restore Cyclin A levels, confirming a lack of effect on cell cycle arrest. For RNA-seq and cytokine arrays, n=3 independent conditions were analyzed. Data are presented as Mean ± S.E.M. Statistical tests: Student’s t-test for (c) and one-way ANOVA for (d), *p<0.05; **p<0.01; ***p<0.001; ***p<0.0001.

Having observed that acetate can alter expression of SASP genes in cells devoid of mitochondria, we sought to examine the role of key mitochondrial metabolic regulators in senescent cells. We first examined the mitochondrial pyruvate carrier (MPC), an obligate hetero-oligomeric complex composed of transmembrane proteins MPC1 and MPC2. The MPC complex imports pyruvate from the cytosol into the mitochondria, serving as a canonical link between glycolysis-derived cytosolic pyruvate and the tricarboxylic acid (TCA) cycle (Fig.2a). We observed that MPC1 protein is upregulated in different types of senescence, including irradiation-induced senescence, doxorubicin-induced senescence, and replicative senescence (Fig. 2b). We also observed significant increases in *MPC1* and 2 in senescent cells at the mRNA level (Fig. 2c).

To further explore the role of MPC in senescence, we pharmacologically inhibited this transporter using small molecule UK5099. Consistent with reduced mitochondrial pyruvate import, UK5099 resulted in a significant decrease in levels of tricarboxylic acid cycle (TCA) metabolites in senescent cells along with increased lactate levels, consistent with a switch towards glycolysis (Fig. 2d).

To comprehensively analyze global gene expression changes induced by MPC inhibition in senescent cells, we performed RNA sequencing. The results revealed that a large set of SASP factors were significantly downregulated following MPC inhibition (Fig. 2e). Similarly, cytokine array revealed that several secreted SASP proteins were downregulated upon MPC inhibition (Fig. 2f). However, there were no differences in the expression of genes associated with proliferation (Fig. 2g). Consistently, we observed no changes in protein expression of p16, p21, cyclin A (Fig. 2h), no changes in EdU incorporation or number of DNA damage foci (Extended data Fig.3a-f). Similar results were observed in irradiation, doxorubicin-induced and replicative senescence (Extended data Fig.3e-j). Furthermore, to confirm these data, we utilized CRISPR/Cas9 gene editing to generate human fibroblasts deficient in MPC1. We found that deletion of MPC1 resulted in a reduction in the mRNA expression of SASP factors *IL8* and *IL6* during senescence (Extended Data Fig.3k and l). These findings demonstrate that inhibiting MPC selectively attenuates the SASP while preserving the cell cycle arrest characteristic of senescent cells.

### Upregulation of Mitochondrial Citrate Carrier (SLC25A1) in senescence modulate the SASP.

**Figure 3:**
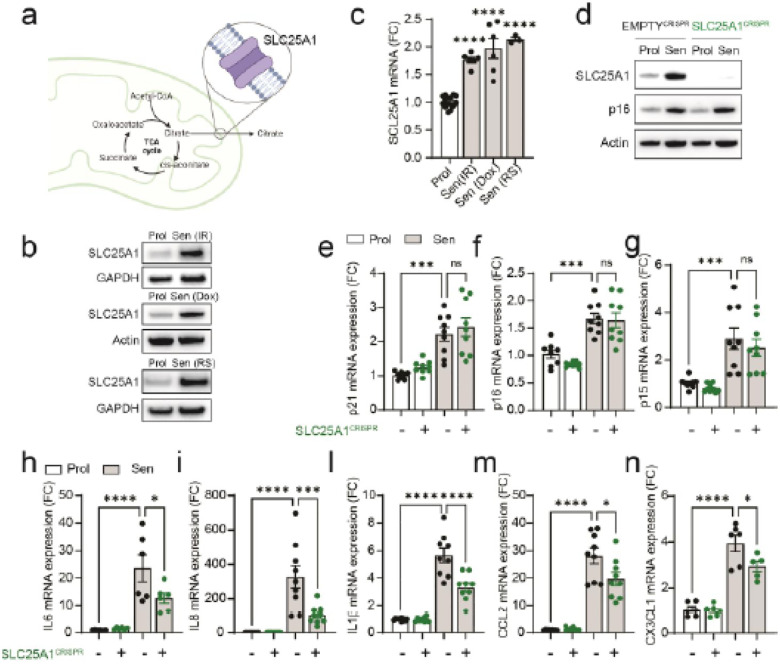
Upregulation of the Mitochondrial Citrate Carrier (SLC25A1) modulates the SASP in senescent cells. **(a)** Schematic illustration of SLC25A1 localization and function within mitochondria. **(b)** Western blot showing increased SLC25A1 protein levels in senescence models: irradiation-induced (Sen(IR)), doxorubicin-induced (Sen(Dox)), and replicative senescence (Sen(RS)). **(c)** mRNA expression of *SLC25A1* in these senescence models compared to proliferative cells. **(d)** Western blot confirming successful CRISPR/Cas9-mediated deletion of *SLC25A1* in proliferative (Prol) and senescent (Sen(IR)) cells, with persistent p16 expression in senescent cells after knockout. **(e-g)** mRNA expression of cyclin-dependent kinase inhibitors (cdki) in different senescence models**. (h-n)** mRNA expression of various SASP components under the same conditions. All values are presented as fold change relative to proliferative cells (Prol) (n=6–9 per condition). Data are shown as Mean ± S.E.M. *One-way ANOVA test: *p<0.05; **p<0.01; ***p<0.001; ***p<0.0001.

Acetyl-CoA produced by mitochondria during the TCA cycle is not able to permeate or otherwise cross the mitochondrial membranes, so it is transported as citrate through the mitochondrial tricarboxylate transporter (Citrate/Isocitrate Carrier) (SLC25A1)^18^. Upon analyzing cells induced into senescence through irradiation, doxorubicin treatment, and replicative exhaustion, we observed a significant increase in SLC25A1 expression at both mRNA and protein levels (Fig. 3b & c). To further investigate the role of SLC25A1 in senescence, we generated SLC25A1 CRISPR-Cas9 knockout cells (Fig. 3d). The absence of SLC25A1 did not affect cell cycle arrest in senescent cells, as indicated by no change in expression of cyclin-dependent kinase inhibitors p21, p16 and p15 (Fig. 3e–g). However, we observed a significant reduction in mRNA levels of several common SASP factors in SLC25A1 knockout senescent cells (Fig. 3h–n).

**Figure 4:**
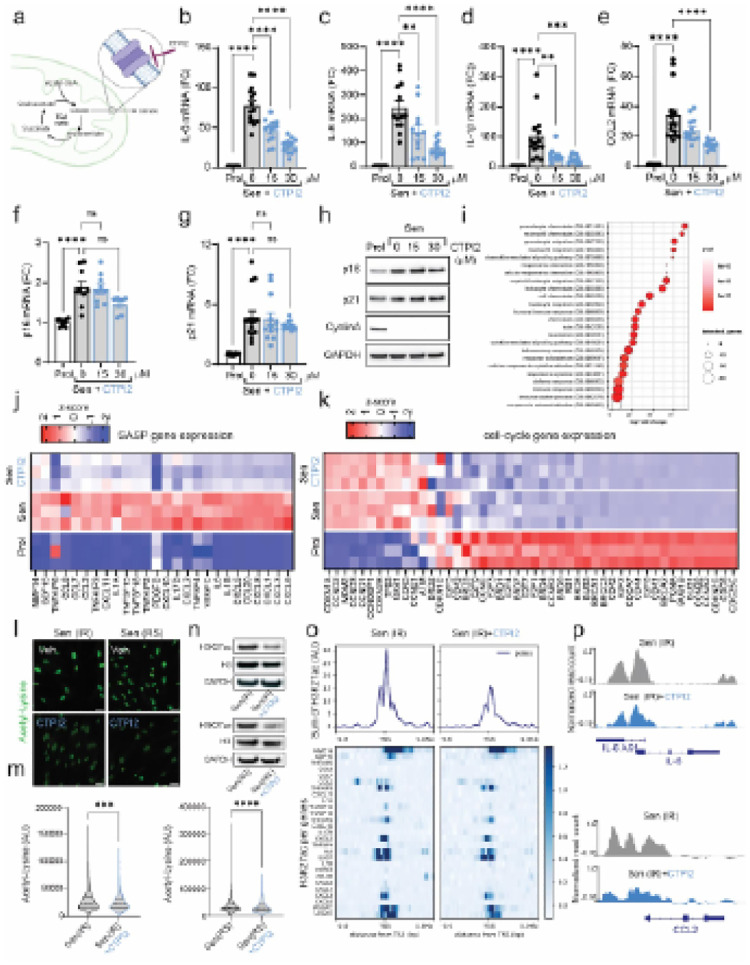
Pharmacological inhibition of Mitochondrial Citrate Carrier (SLC25A1) with CTPI2 reduces the SASP in senescent cells. **(a)** Schematic illustrating experimental approach. **(b-g**) mRNA expression levels of SASP components **(b-e)** and cyclin-dependent kinase inhibitors (cdki) **(f-g)** in senescent cells (Sen(IR)) treated with two doses of CTPI2 (15 and 30 μM), expressed as fold change relative to proliferative cells (Prol) (n=6–12). **(h)** Western blot analysis showing that CTPI2 treatment does not alter p16 or p21 expression, nor does it restore Cyclin A levels, indicating that the cell cycle arrest is maintained. **(i)** Gene ontology pathways of genes downregulated by CTPI2 compared to untreated senescent cells. **(j-k)** Column-clustered heatmaps showing SASP factors **(j)** and cell cycle-associated genes **(k)** differentially expressed in senescent cells and downregulated by CTPI2 treatment (color intensity represents column Z-scores, where red indicates high expression and blue indicates low expression; n=3 independent experiments). **(l)** Representative immunofluorescence images of nuclear acetylated lysine (green) in two models of senescence treated with or without CTPI2. Scale bar = 50 μm. **(m)** Quantification of nuclear acetyl-L-lysine integrated density *per* cell (n=3 independent experiments). **(n)** Western blot showing reduced histone acetylation (H3K27ac) after CTPI2 treatment across two different senescence inducers. **(o)** Metaplot (top) and heatmap (bottom) of SASP genes showing reduced histone H3K27 acetylation (H3K27ac) levels normalized by histone H3 around the transcription start sites (±5 kb) in senescent cells (Sen(IR)) after CTPI2 treatment (data averaged from n=3 independent experiments)**. (p)** Browser tracks of *IL6* and *CCL2* genes depicting reduced H3K27ac levels following CTPI2 treatment. Data are shown as Mean ± S.E.M. *One-way ANOVA or Student’s t-test: *p<0.05; **p<0.01; ***p<0.001; ***p<0.0001.

Following these observations, we tested the impact of small molecule CTPI2, a third generation SLC25A1 inhibitor with high binding activity and specificity^19^. Treatment of senescent cells with CTPI2, resulted in a dose-dependent reduction of SASP components at the mRNA level (Fig.4b–e) but no change in the expression of p21 and p16 at both the mRNA and protein level (Fig.4f–h), cyclin A protein levels (Fig.4h). Similarly, we observed decreases in secretion of common SASP factors by cytokine array (Extended data Fig.4a). To further understand how CTPI2 impacts on gene expression in senescent cells, we performed RNA sequencing. Gene ontology analysis revealed that CTPI2 treatment in senescent cells primarily downregulated pathways associated with inflammatory processes (Fig.4i) and common SASP genes were also downregulated in senescent cells treated with CTPI2 (Fig.4j). Notably, no changes were observed in the expression of genes associated with the cycle-cycle that are typically modulated in senescent cells (Fig. 4k). Similarly, other features of senescence such as loss of nuclear HMGB1, DNA damage foci (gH2A.X) and % of Ki67 cells were also unaffected by CTPI2 treatment (Extended Data Fig.4b and c). Consistent with our hypothesis that inhibition of SLC25A1 regulates the SASP via histone acetylation, we found that CTPI2 decreased pan-acetyl-L-lysine staining in the nuclei (Fig.4l and m) and decreased H3K27ac levels relative to total H3 (Fig.4n). ChIP-seq analysis with an H3K27ac-specific antibody revealed that CTPI2-treated senescent cells exhibit reduced histone acetylation at SASP gene loci identified as downregulated in RNA sequencing (Fig.4o). Representative examples of two SASP components, *IL6* and *CCL2*, are shown in Fig.4p.

The effect of CTPI2 on SASP was also observed when senescence was induced by the chemotherapy drug doxorubicin (Extended Data Fig.5a). To determine if CTPI2 retains its SASP-inhibiting properties after the onset of senescence, we treated cells either immediately after irradiation (day 2) or when the SASP is already induced (day 8). CTPI2 consistently reduced SASP mRNA expression at both time points, without affecting p16 or p21 expression (Extended Data Fig.5b). Similarly, treatment of replicatively senescent cells with CTPI2 significantly decreased expression of SASP factors without affecting p21 or p21 expression (Extended Data Fig.5c). Altogether, these data suggest that SLC25A1 regulates the SASP without affecting the senescence-associated cell-cycle arrest.

Given the key role of SLC25A1 in mitochondrial metabolism, we tested whether CTPI2 impacts on mitochondrial function. RNA-sequencing analysis showed no changes in OXPHOS gene expression in treated senescent cells compared to senescent controls (Extended Data Fig.6a). Using Seahorse flux analysis, we observed that CTPI2 increased maximal mitochondrial respiration rates in senescent cells but did not affect ATP production or coupling efficiency (Extended Data Fig.6b-f). Furthermore, CTPI2 did not change mitochondrial ROS production or the appearance of the mitochondrial network (Extended Data Fig.6g-i). However, CTPI2 significantly reduced the levels of certain TCA metabolites, including isocitrate, cis-aconitate, and citrate (Extended Data Fig.6j). Collectively, these findings show that SLC25A1 inhibition by CTPI2 suppresses the SASP in senescent cells by modulating histone acetylation, without affecting other hallmarks of senescence or impacting mitochondrial respiration and dynamics.

### Mitochondria-derived Citrate drives the SASP via ACLY

**Figure 5:**
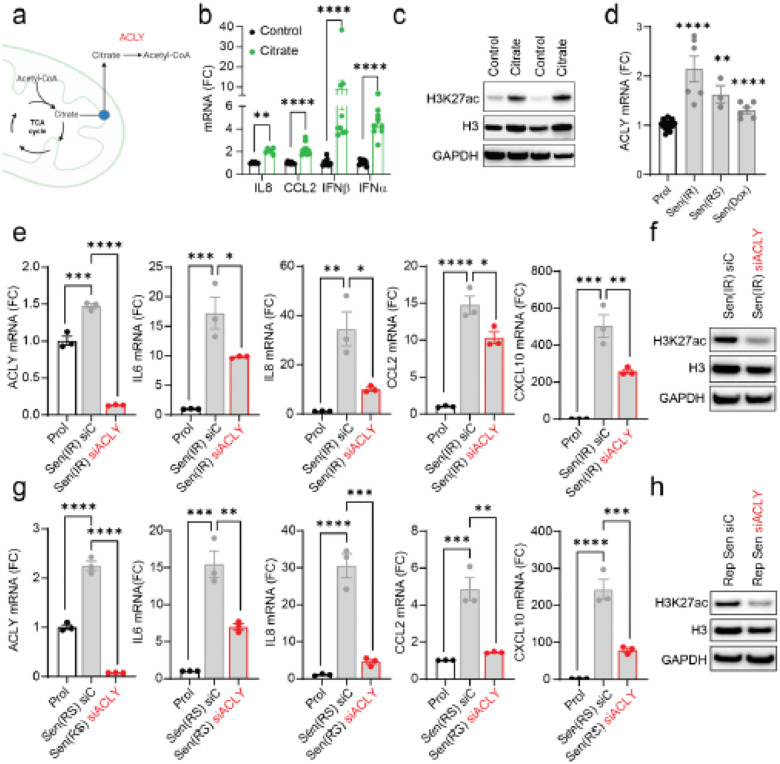
Mitochondria-derived Citrate drives the SASP through ACLY activity. **(a)** Schematic representation illustrating the export of citrate from mitochondria and conversion to Acetyl- CoA. **(b)** Relative mRNA expression of SASP components in proliferative cells treated with 10 mM citrate (Citrate) compared to untreated proliferative cells (Control), shown as fold change. **(c)** Western blot analysis of histone H3 acetylation (H3K27ac) and total histone H3, demonstrating increased histone acetylation in citrate-treated cells. **(d)** Relative mRNA expression of ACLY in cells undergoing senescence induced by various stimuli. **(e)** mRNA expression in irradiation-induced senescence (Sen(IR)) showing successful ACLY knockdown by siRNA and subsequent effects on SASP gene components, expressed as fold change relative to proliferative cells (Prol)**. (f)** Western blot analysis showing reduced histone H3 acetylation (H3K27ac) following ACLY silencing in Sen(IR) cells. **(g)** mRNA expression in replicative senescence (Sen(RS)) showing effective ACLY knockdown by siRNA and effects on SASP gene expression, presented as fold change relative to proliferative cells (Prol). **(h)** Western blot analysis indicating decreased histone H3 acetylation (H3K27ac) in Sen(RS) cells following ACLY silencing. Data are represented as Mean ± S.E.M. Statistical analysis was performed using Student’s t-test for **(b, d**) and one-way ANOVA for **(e, g)**. *p < 0.05; **p < 0.01; ***p < 0.001; ****p < 0.0001.

Given our results showing that expression of SCL25A1 increases in senescence and its inhibition reduces the SASP and citrate levels, we hypothesized that increased cytosolic citrate in senescent cells could drive the SASP. Citrate, when in the cytosol, can be converted into acetyl-CoA via the enzyme ATP-citrate lyase (ACLY) (Fig.5a).

To further test the role of citrate in the regulation of the SASP, we supplemented proliferating fibroblasts with citrate and observed an increased expression of SASP genes (Fig.5b) associated with elevated H3K27ac levels relative to total H3 (Fig.5c). We also found that mRNA expression of ACLY was increased in senescent cells induced by irradiation, doxorubicin and replicative exhaustion (Fig. 5d).

To determine the role of ACLY in senescence, we silenced its expression using siRNA which led to reduced expression of SASP components and decreased H3K27ac protein expression relative to total H3 in both irradiated and replicatively senescent cells (Fig.5e–h). Altogether, these data support a model in which mitochondria-derived citrate and its subsequent conversion to acetyl-CoA via ACLY contributes to the SASP via histone acetylation.

### Targeting Mitochondrial Citrate Carrier improves health metrics

**Figure 6:**
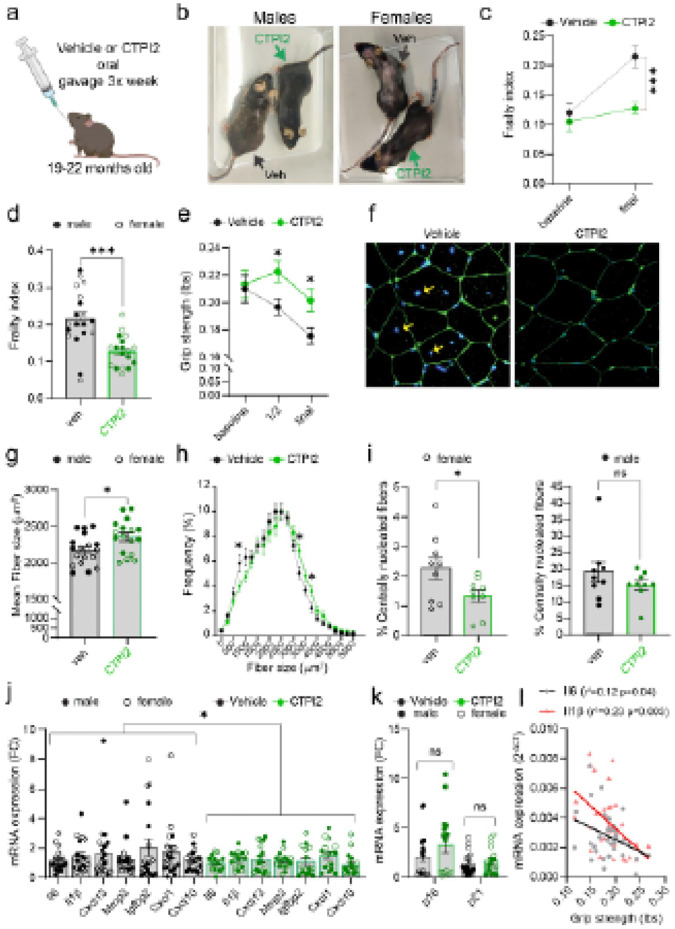
Pharmacological inhibition of Mitochondrial Citrate Carrier (SLC25A1) during aging improves frailty and muscle function. **(a)** Schematic representation of the *in vivo* experimental procedure detailing CTPI2 treatment in aged mice. **(b)** Representative images of male and female mice treated with either vehicle or CTPI2, as indicated by the green arrows. **(c)** Frailty index scores at baseline (0 months) and after 3 months of treatment (final) in vehicle (n = 19) and CTPI2-treated (n = 19) mice (males and females combined). **(d)** Histogram of frailty index scores after 3 months of treatment, showing individual values for vehicle (n = 19) and CTPI2-treated (n = 19) mice. Males are denoted by filled black dots, and females by open black dots. **(e)** Forelimb grip strength measured at baseline, 1.5 months, and 3 months (final) in each group (n = 19 per group). **(f)** Representative images of WGA staining, marking the membrane of cross-sectional myofibers. **(g)** Quantification of mean cross-sectional myofiber area *per* mouse. **(h)** Distribution of cross-sectional myofiber area *per* mouse. **(i)** Percentage of centrally nucleated fibers per mouse, presented separately for females (open dots, left panel) and males (filled dots, right panel). **(j-k)** mRNA expression of SASP components **(j)** and cell cycle inhibitors (p16, p21) **(k)** in quadriceps muscle from male and female mice, expressed as fold change relative to vehicle-treated mice**. (l)** Correlation curves of Il1b or Il6 expression with forelimb grip strength for each mouse. Data are presented as Mean ± S.E.M. Statistical analysis performed using Student’s t-test. *p < 0.05; **p < 0.01; ***p < 0.001; ****p < 0.0001. Vehicle: n = 10 males and 9 females; CTPI2: n = 9 males and 10 females. Males are represented by filled dots, and females by open dots.

Our *in vitro* data suggests that CTPI2 could serve as a promising therapeutic strategy to target senescent cells in aging by selectively suppressing the SASP. To further explore this possibility, we first isolated stromal cells from young (3 months) and old (24 months) hearts and kidneys and treated them *in vitro* with CTPI2 (Extended Data Fig.7a and b). Aged stromal cells exhibited elevated expression levels of p16 and SASP components compared to young cells (Extended Data Fig.7c-f). Notably, treatment with CTPI2 significantly diminished the expression of SASP factors in aged stromal cells while leaving p16 expression unaltered (Extended Data Fig.7c-f). Encouraged by these findings, we administered CTPI2 directly to aged mice (from 19-= to 22 months old) via oral gavage three times *per* week for a duration of three months (Fig.6a) and examined several healthspan parameters.

CTPI2-treated mice (both male and female) exhibited a more lustrous and uniform fur coat, reduced alopecia, and less fur graying compared to vehicle (Fig.6b), associated with delayed onset of frailty, using a 31-parameter frailty index^20^ (Fig.6c and d) and increased forelimb strength (Fig.6e). Histological analysis of muscle tissue revealed that CTPI2 treatment led to larger cross-sectional myofiber areas, suggesting delayed age-related muscle atrophy (Fig.6f–h) and significantly reduced the proportion of centrally nucleated fibers in female mice with a similar trend in males (Fig.6i). Despite these improvements in musculoskeletal phenotypes, CTPI2 treatment did not improve spine and femur bone microarchitecture (Extended data Fig.8a).

qPCR analysis of muscle tissue showed that CTPI2 treatment reduced SASP gene expression without affecting p16 or p21 levels (Fig.6j and k). Interestingly, we found a negative correlation between expression of pro-inflammatory factors *Il1β* and *Il6* and grip strength, suggesting that CTPI2 may enhance muscle function by reducing inflammation (Fig.6l). CTPI2 treatment also reduced muscle lipid accumulation, with sex-specific differences. In males, lipid size decreased, while in females, total lipid area decreased (Extended data Fig.8b-d).

We examined the effects of CTPI2 treatment on cardiac tissue and found no reduction in classical SASP factors or senescence markers (p16 and p21) in whole heart samples (Extended Data Fig.9a-b). However, since senescent cardiomyocytes express a distinct SASP profile during aging, CTPI2 significantly decreased cardiomyocyte-specific SASP components *Gdf15* and *Edn3* (Extended Data Fig.9c). This was accompanied by reduced cardiomyocyte hypertrophy, a hallmark of cardiac aging, suggesting an overall improvement in cardiac function (Extended Data Fig.9d). In contrast to whole heart tissue, isolated cardiac stromal cells showed reduced expression of classical SASP factors (*Ccl2, Il6, Cxcl1, Il1α*) without affecting p16 or p21 expression (Extended Data Fig.9e-f). Consistent with a role for the SASP in the recruitment of immune cells^21,22^, we found that CTPI2 significantly reduced the levels of pro-inflammatory CCR2-positive macrophages in the heart, particularly in female mice (Extended Data Fig.9g-h). CTPI2 also reduced pro-inflammatory factors in the stromal cell-enriched fraction of the liver without altering p21 or p16 expression, further highlighting its anti-inflammatory effects (Extended Data Fig.9i-j). However, no reduction in SASP markers was observed in the total liver tissue (Extended Data Fig.9k-l), suggesting that the effect of CTPI2 may be more pronounced in stromal cells compared to hepatocytes.

In summary, our data demonstrate that CTPI2 treatment in aged mice improves healthspan by reducing the SASP across multiple organs, offering a promising avenue for therapeutic intervention in aging.

## Discussion

The regulation of the epigenome plays a critical role in shaping various aspects of the senescence phenotype, including cell-cycle arrest and the SASP^23^. For example, the histone lysine demethylase KDM4A enhances SASP expression by reducing the repressive H3K9me3 mark on SASP genes, while MLL, a histone methyltransferase, promotes the SASP by adding the activating H3K4me3 mark^24^. Distal gene enhancers marked by H3K27ac also contribute to SASP activation^25,26^.

Acetyl-CoA, the primary acetyl donor for histone acetylation, is crucial in regulating chromatin structure. It is derived from mitochondria in the form of citrate, which is transported via the SLC25A1 tricarboxylate transporter^18^. Enzymes like ATP-citrate lyase (ACLY) convert this citrate into acetyl-CoA outside the mitochondria, enabling histone acetylation, including the H3K27ac mark that promotes SASP gene activation^27^. Recent studies, including our findings, show that inhibiting ACLY in senescent cells reduces both histone acetylation and SASP expression^28^.

Our study confirms the critical role of mitochondria in the epigenetic regulation of the SASP. By clearing mitochondria via Parkin-mediated mitophagy, we observed a reduction in H3K27 acetylation at key SASP gene promoters, along with a decrease in SASP expression. While we focused our analysis on H3K27 acetylation, we observed that other histone acetylation marks are present at mitochondria-dependent SASP genes, during senescence. These findings indicate that mitochondrial function is closely linked to epigenomic changes driving the SASP.

Additionally, our data demonstrate that inhibiting SLC25A1 reduces H3K27ac levels and SASP gene expression, while citrate supplementation increases both. This aligns with previous reports linking citrate metabolism to SASP regulation^28^. Furthermore, we show that inhibiting ACLY similarly decreases SASP expression, reinforcing the role of citrate-derived acetyl-CoA in histone acetylation. The relationship between the metabolic-epigenetic regulation of the SASP and our recent findings that mitochondrial dysfunction during senescence drives cytosolic leakage of mitochondrial DNA^29^, RNA^30^ and chromatin fragments^31^ also contributes to the SASP remains unclear. Elucidating how these distinct factors interact to regulate the SASP will be key for gaining a more comprehensive understanding of mitochondrial involvement in this process.

Our findings have important therapeutic implications for aging. Pharmacological inhibition of SLC25A1 using CTPI-2, a selective and potent inhibitor^32^, improved frailty and muscle function in aged mice, while also reducing inflammation in skeletal muscle, liver, and heart. Although CTPI-2 has previously been shown to improve metabolic conditions like steatohepatitis^33^, its effects in the context of aging had not been explored until this study.

In conclusion, our study supports the hypothesis that mitochondrial metabolic changes influence SASP regulation and can be targeted for senotherapeutic interventions. We propose that mitochondrial-derived citrate, regulated by proteins like MPC and SLC25A1, fuels ACLY-mediated histone acetylation of SASP genes, driving age-related inflammation.

## Materials And Methods

### Cell culture and treatments

Human embryonic lung MRC5 fibroblasts (ATCC) and IMR90 fibroblasts (ATCC) were cultured in Dulbecco’s modified Eagle’s medium (Sigma-Aldrich, D5796) supplemented with 10% heat-inactivated fetal bovine serum (FBS), 100 U ml−1 penicillin, 100 μg ml−1 streptomycin and 2 mM l-glutamine. The cultures were maintained at 37°C in an atmosphere of 5% CO2. MRC5 fibroblasts were grown under atmospheric oxygen conditions, while IMR90 fibroblasts were cultured under low-oxygen (3%) conditions.

For lentiviral transduction HEK293T cells (ATCC) were used, cultured in DMEM without antibiotic and supplemented with 10% heat-inactivated fetal bovine serum (FBS) and 2mM L-glutamine.

Stress-induced senescence was triggered by exposing cells to 20 Gy of X-ray irradiation and collected between 10- and 12-days post-irradiation. Chemotherapy-induced senescence was performed by treated cells with 250nM of doxorubicin (MedChemExpress, HY-15142) for 24h and collected 12 days after treatment. Replicative senescence was performed through serially passaging until the cells reached their replicative limit. Senescence was verified through the presence of p16 and p21, the absence of proliferation markers Ki-67 or EdU incorporation, and the expression of SASP gene.

For chronic acetate treatment, MRC5 or Parkin IMR90 cells were treated with 20mM of Sodium Acetate solution (Sigma, S7899) for 10 to 12 days, with media refreshing every 48–72h.

For chronic Citrate treatment, MRC5 were supplemented with 10mM of Sodium Citrate (Sigma, W302600) for 10 to 12 days, with media refreshing every 48–72h.

For CIC and MPC pharmacological inhibition, MRC5 fibroblasts were irradiated with 20 Gy X-ray irradiation and treated with CTPI2 (Selleckchem, S2968) or UK5099 (Sigma, PZ0160) at the indicated concentrations (15 or 30μM for CTPI2 and 100μM for UK5099). CTPI2 and UK5099 were added one day after irradiation and maintained in the cell culture medium for 12 days (refreshed every 48–72 h).

### Parkin-mediated mitochondria clearance

Parkin-mediated mitochondrial clearance was performed as previously described. In summary, proliferating or irradiated Parkin-overexpressing IMR90 fibroblasts were treated with 12.5μM CCCP (Sigma-Aldrich, C2759) one day post-irradiation (D1) for a duration of 48 hours, with CCCP being replenished every 24 hours (D1, D2). Acetate was added at D3 when mitochondria were cleared, and cells were harvested at D12 (media refreshed every 48–72h).

### CRISPR–CAS9-based genome editing

The following plasmids were used:

hSLC25a1 CRISPR (sgRNA #231; Vector-Builder, VB900058–0699rvd), hMPC1 CRISPR (5’-CACCGGGGCTACTTCATTTGTTGCG-3’ AND 5’-AAACCGCAACAAATGAAGTAGCCCC-3’).

For lentiviral transduction, HEK293FT cells were transfected with the plasmids above together with the packaging and envelope plasmids VSVG and Gag-Pol (Sigma-Aldrich) using Lipofectamine 3000 (Invitrogen, L3000015) according to the manufacturer’s instructions. Then, 2 days later, the supernatant from the transfected HEK293FT cells containing viral particles was filtered using a 0.45μm pore PVDF filter, mixed with 10 μg/ml of polybrene and used to infect the cells of interest. After infection, cells were selected for successful CRISPR–Cas9 deletion using the following antibiotics: 1μg/ml of puromycin.

### siRNA

siACLY (Sigma, SASI_Hs01_00239323) and siScramble (Sigma, SIC001) were used for the experiments.

For senescence induced by irradiation: Cells were firstly transfected in T75 ask with 30nM final of siRNA using the DharmaFECT 2 transfection reagent (Horizon, T-2002–03) at a ratio of 0.3/100μl of transfection media for 24h. The day after cells were irradiated with 20Gy X-ray, and seed at D1 post irradiation in 6 wells plates. Cells were transfected a second time at D8 post irradiation with 30nM final of siRNA using the same transfection reagent for 24h. Cells were finally collected at D10 post irradiation for analysis.

For replicatives senescence: Replicatives senescent cells were transfected twice D1 and D4 with 30nM final of siRNA using the DharmaFECT 2 transfection reagent (Horizon, T-2002–03) at a ratio of 0.3/100μl of transfection media for both 24h and collected at D6 for analysis.

### TCA metabolite

Concentration of TCA analytes were measured by gas chromatograph mass spectrometry (GC/MS) as previously described with a few modifications. Briefly, cell pellet was lysed in 50 l 1X PBS after adding 20μl of internal solution containing U-^13^C labeled analytes. The proteins were removed by adding 300μl of chilled methanol and acetonitrile solution to the sample mixture. After drying the supernatant in the speed vac, the sample was derivatized with ethoxime and then with MtBSTFA + 1% tBDMCS (N-Methyl-N-(t-Butyldimethylsilyl)-Trifluoroacetamide + 1% t-Butyldimethylchlorosilane) before it was analyzed on an Agilent 5977B GC/MS (Santa Clara, CA) under electron impact and single ion monitoring conditions. Concentrations of lactic acid (m/z 261.2), fumaric acid (m/z 287.1), succinic acid (m/z 289.1), ketoglutaric acid (m/z 360.2), malic acid (m/z 419.3), aspartic acid (m/z 418.2), 2-hydroxyglutaric acid (m/z 433.2), cis aconitic acid (m/z 459.3), citric acid (m/z 591.4), and isocitric acid (m/z 591.4), glutamic acid (m/z 432.4) were measured against a 12-point calibration curves that underwent the same derivatization.

### Metabolomic by Mass spectrometry

Metabolomics experiments for UK5099 treatment were performed as described below. Cells were seeded into 6-well plates (5 × 10^5^−1.5 × 10^6^ cells per ml, triplicate wells per condition) in complete DMEM medium and were allowed to adhere overnight for proliferatives controls, or cultured and treated as described previously for Sen(IR) and Sen(IR)+UK5099. Cells were washed with PBS, and the relevant experimental media were added for the stated times. Duplicate wells were used for cell counting: cell counts were used to normalize the volume of lysis solvent prior to metabolite extractions (2 × 10^6^ cells per ml). Cells were washed quickly in PBS, then ice-cold lysis solvent (methanol 50%, acetonitrile 30%, water 20%) was added and cells were scraped on ice. Lysates were transferred to 1.5-ml tubes on ice, vortexed and then centrifuged at 15,000 r.p.m. at 4 °C for 10 min. Supernatants were collected and stored at −80 °C for LC–MS analysis. The flow rate was set to 200 μl per min and the injection volume was 20 μl. The separation was done using an isocratic program of 80% A and 20% B, with a total run time of 3 min. The Exactive mass spectrometer was operated in full-scan mode over a mass range of 50–800 *m/z* at a resolution of 50,000 in positive mode.

### ChIP-sequencing

Chromatine ImmunoPrecipiation (ChIP) was performed as describe before^34^. Cells were formalin fixed for 10 minutes in 4%PFA, then lysed and chromatin was sheared to between 250–500bp as confirmed by agarose gel electrophoresis in size using sonication (Biorupter pico, Ref B01060010). Protein G Dynabeads (Invitrogen, Ref 1000D) were prepared with 5μg of primary antibody of interest: Histone H3 (Abcam ref ab1791), Histone H3K27ac (Abcam, ref ab4729). 1.7μg chromatin as quantitated by qubit dsDNA HS Kit (Invitrogen Ref Q32854) were used per ChIP and IP was allowed overnight 4°C while inverting. After washing steps, DNA was eluted using phenol chloroform isoamyl alcohol (Thermo Scientific, Ref J62336) isolation and ethanol precipitation then quantitated using qubit dsDNA HS Kit (Invitrogen Ref Q32854) and submitted to Sanford Burnham Prebys genomic core for library preparation and sequencing on Element Biosciences AVITI sequencer.

ChIP-seq was performed as following described: Library preparation of ChIP DNA was performed with the Watchmaker DNA Library Prep Kit (Watchmaker Genomics, Cat: 7K0103) with xGEN Stubby adaptors (IDT, Cat: 10005924) and xGEN 10nt UDI Primers (IDT, Cat: 10008052). Libraries were sequenced (2×76bp) with the Element Biosciences AVITI Sequencing platform using the AVITI 2×75 High Output Cloudbreak Freestyle Kit (Element Biosciences, Cat: 860–00015).

### Generation ChIP-seq analysis files

H2K27ac and H3 ChIP-seq samples were processed with nf-core chipseq nextflow pipeline version 2.0.0 [PMID: 32055031] using singularity containers and parameters “--aligner bowtie2 --read_length 75--genome GRCh38”. H3K27ac bigwig signal les were normalized by H3 signal using Deeptools bigwigCompare version 3.5.5 [PMID: 24799436] with parameters “–operation subtract --binSize 10”.

### ChIP-seq databases analysis, track visualization and Heatmaps generation.

Already published ChIP-seq data from GSE106146, GSE56307, GSE74238 and GSE103590, along with new generated ChIP-seq experiment available in GSE279410. The new ChIP-seq data represent the average of 3 replicated for each condition.

Specific genes track visualization was performed on Integrative Genome Viewer. And metaplot and heatmap were generated on Galaxy website, using deepTools, computeMatrix and plotHeatmap.

### MitoSOX

Cells were grown in 96 well dark plate for fluorescence reading. The experiment was conduct at D12 post irradiation and proliferatives cells were seeded in the plate the day before the measurement. The MitoSOX Red Mitochondrial Superoxide indicator (Thermo, M36008) was used for the experiment. Cells were stained for 10 minutes with 5μM of MitoSOX in serum free media. After two PBS wash, fluorescence intensity was quantified in PBS every two minutes for 50 minutes at 37°C by Varioskan Lux 35 (Life Technologies).

### Seahorse analysis

Cellular oxygen consumption rate (OCR) and extracellular acidification rate (ECAR) were measured using Agilent Seahorse XFe96 Analyzer with the Mito Stress Test Kit (Agilent, 103015–100), according to manufacturer’s instructions. Cells were seeded at a concentration of 5000 cells per wells and irradiated the day after. The experiment was running at D12 post-irradiation and proliferatives cells were seeded at the day before. The assay medium was prepared by supplementing Seahorse XF DMEM with 1mM pyruvate, 2mM glutamine and 10mM glucose. CTPI2 inhibitors were maintain at the concentrations mentioned during the experiment. For the mitochondrial activity the following compound were added to the test: Oligomycin 1.5μM, FCCP 1μM and Rotenone/Antimycin A 0.5μM.

### Multiplex Immunoassay for Cytokine Concentration in Condition Media

24h FBS free conditioned media were generated from 80 000 cellules per condition, collected and centrifuged at 2,000 x rpm at 4°C for 5 minutes to remove cell debris. The supernatant was aliquoted and stored at −80°C until use. On the day of the assay, each sample aliquot was thawed on ice and processed. Cytokine concentrations were measured simultaneously using Luminex xMAP^®^ Multiplexing Technology with antibody kits from Bio-Techne (FCSTM18B, LXSAHM). Samples were diluted 1:2 in the dilution buffer provided with each kit, and the recommended protocols were followed. Minimum fluorescence intensity was measured using the Luminex Intelliflex system. A 5-parameter logistic (5PL) regression model was applied to each cytokine standard curve to calculate cytokine concentrations using Quantist Software (Bio-Techne). Final cytokine abundance was expressed as pg/ml.

### Western blotting

Cells were lysed in lysis buffer (RIPA: 150 mM NaCl, 1% NP40, 0.5% sodium deoxycholate, 0.1% SDS, 50 mM Tris pH 7.4, 1× phosphatase and protease inhibitors cocktail in H_2_O) and the protein concentration was determined using the Bio-Rad protein assay (Bio-Rad, reagent A, 500–0113; reagent B, 500–0114; reagent C, 500–0115). Proteins were deposed by equal amount on each well and separated by molecular weight on NuPAGE 4 to 12% Bis-Tris gels (Invitrogen, NP0323). Proteins were secondary blotted on PVDF membrane using Power Blotter XL machine (Invitrogen, PB0010) and Power Blotter Select Transfer Stacks, PVDF, regular size (Invitrogen, PB5310). Membranes were blocked with TBS-Tween (TBS-T) blocking buffer (5% milk powder, 0.05% Tween-20 in TBS) and incubated with primary antibodies at 4 °C overnight (a list of the antibodies Supplementary Table 2). After washes in TBS-T, the membranes were incubated with a peroxidase-conjugated secondary antibody for at least 1h at room temperature. The membranes were then incubated with either SuperSignal^™^ West Pico PLUS Chemiluminescent Substrate (Thermo Scientific, 34577) or the KwikQuant Western blot detection kit (Kindle Bioscience, R1100) according to manufacturer’s instructions, and visualized using iBright 1500 system from Invitrogen.

### RT-qPCR

Total RNA was extracted using QIAshredder column (Qiagen, 79656) following by RNeasy Mini Kit (Qiagen, 74106). mRNAs were quantified by spectrophotometry (NanoDrop One, Thermo Fisher Scientific). cDNAs were synthesized with the MultiScribe^™^ reverse transcriptase (High-Capacity cDNA Reverse Transcription Kit, Applied Biosystem). PCR was performed for 40 cycles with TaqMan Probes (IDT) in Perfecta qPCR Tough mix (Quantabio95112.012) and run on CFX Opus 384 Real Time PCR Detection System (BioRad). Different Probes used are listed in Supplementary Table 1. TBP for human cells and HPRT for mice sample were used as a reference gene for normalization and relative gene expression, compared to the control group, was calculated using the comparative cycle threshold (CT) method (2-ΔΔCT).

### RNA-sequencing

RNA-sequencing were performed by Azenta Life Sciences. The sequencing configuration was Illumina, 2×150bp, with around 30 million paired end reads per sample.

Sequence reads were trimmed to remove possible adapter sequences and nucleotides with poor quality using Trimmomatic v.0.36. The trimmed reads were mapped to the Homo sapiens GRCh38 reference genome available on ENSEMBL using the STAR aligner v.2.5.2b. The STAR aligner is a splice aligner that detects splice junctions and incorporates them to help align the entire read sequences. BAM les were generated as a result of this step. Below are the statistics of mapping the reads to the reference genome.

Unique gene hit counts were calculated by using feature Counts from the Subread package v.1.5.2. The hit counts were summarized and reported using the gene_id feature in the annotation le. Only unique reads that fell within exon regions were counted. If a strand-specific library preparation was performed, the reads were strand-specifically counted.

After extraction of gene hit counts, the gene hit counts table was used for downstream differential expression analysis. Using DESeq2, a comparison of gene expression between the customer-defined groups of samples was performed. The Wald test was used to generate p-values and log2 fold changes. Genes with an p-value < 0.05 and absolute log2 fold change > 0.5 were called as differentially expressed genes for each comparison.

Data available in the GEO repository: GSE279411.

### Immunocytochemistry

Cells were cultured on coverslip and fixed for 10 min using either 4% paraformaldehyde (PFA) in PBS for usual staining or 4% PFA with 0.2% of glutaraldehyde for mitochondrial network staining. After 3 PBS washes, cells were blocked and permeabilized for at least 1h at room temperature (RT) with blocking buffer (PBS 0.3% Tritonx100, 5% BSA and 1/60e normal goat serum). Cells were incubated with primary antibodies overnight at 4°C in humid chamber. After PBS washes, cells were incubated with secondary antibodies for at least 1h at RT. After final washes, coverslips were mounted onto glass microscope slides with ProLong Gold Antifade Mountant with DAPI (Invitrogen). A list of the antibodies used is provided in Supplementary Table 2.

Pictures were taken with Leica widefield microscope DMi8 either at x20 or x63 magnification. Images were quantified using ImageJ software.

### Immunohistochemistry

Formalin-fixed paraffin-embedded tissue sections (5 μm) were deparaffinized in xylene (2 times for 5 min each) and hydrated using sequentially 5 minutes batch of 100% ethanol (twice), 90% ethanol, 70% ethanol and distilled water (twice). Antigen retrieval was performed by heating the sections to 98 °C in citrate buffer at pH 6.0 for 15 min. The slides were allowed to cool down for 30 min and were then rinsed in PBS twice for 5 min. To avoid nonspecific binding, tissue sections were blocked for at least 30min at RT in the blocking solution (PBS, 0.1%BSA with 1:60 of normal goat serum). For membrane staining, WGA was applied for 30 min at RT and wash 3 times with PBS. Primary antibodies were incubating in blocking solution overnight at 4°C in humid chamber. After several PBS washes, tissue sections were incubating with secondary antibodies for at least 1h at RT. Finally, sections were mounted with ProLong Gold Antifade Mountant with DAPI (Invitrogen). A list of the antibodies used is provided in Supplementary Table 2.

Pictures were taken with Leica widefield microscope DMi8 either at x20 or x40 magnification. Images were quantified using ImageJ software.

### Mouse models and treatments

All animal experiments were performed according to protocols approved by the Institutional Animal Care and Use Committee (IACUC) at Mayo Clinic. Male and female aged wild-type C57BL/6 mice (aged 19 months) were acquired from the National Institute on Aging (NIA) and were maintained in a pathogen-free facility under a 12 h–12 h light–dark cycle at 23–24 °C with free access to regular chow and water. The mice were housed in same-sex cages in groups of 5. The animals were randomly assigned into the vehicle or treatment group. Mice were gavaged with 30mg/kg of CTPI2 (MedChem Express, HY-123986) diluted in Corn Oil 3 times a week for 3 months (from 19 months to 22 months old), at which point the animals were euthanized and tissues were collected for analysis. Frailty assessment was conducted before and after the 3 months treatments. Grip strength was asset before, at mid-point (1,5 months) and after 3 months of treatment. The mice were euthanized and considered to be dead if they met humane end points.

### Stromal cells isolation

Hearts, livers, and kidneys were harvested from mice after intraventricular perfusion of 10 ml PBS, minced with scalpels and digested with Liberase TM (Roche) diluted in RPMI1640 (GIBCO) as described previously^21^. Briefly, digestion of tissue fragments was performed by two successive incubations for 10 min with enzymatic solution at 37°C under shaking and stopped by the addition of heat-inactivated fetal bovine serum (Gibco). Single cell suspensions were obtained by filtrations on 100 μm and 40 μm cell strainers (BD Falcon). Cells were either pellet and lysed for RNA extraction or seeded for *in vitro* experiment.

### Frailty measurements

Frailty will be assessed based on a 31-items performance-based frailty index that reflects on clinical signs of deterioration in mice, as described before. These clinical assessments include evaluation of integument, the musculoskeletal system, the vestibulocochlear/ auditory systems, the ocular and nasal systems, the digestive system, the urogenital system, the respiratory system, signs of discomfort, body weight, and body surface temperature. The severity of each parameter will be rated as follows: a score of 0 will be given for mice displaying no sign, 0.5 will be given for mild deficits, and a score of 1 will be given if severe deficits are observed.

### Skeletal imaging

All bone imaging and analysis was performed in a blinded manner. Quantitative analysis of the lumbar spine (L4–L6) and distal femoral metaphysis were performed using the Viva Scan 40 μCT scanner (Scanco Medical AG, Basserdorf, Switzerland) with the following parameters: 55kVp, 145mA, high resolution, 21.5 diameter, 10.5 μm voxel size, 300 ms integration time. Using two dimensional (2D) data from scanned slices, 3D analysis was used to calculate morphometric parameters at both the lumbar spine (200 slices) and distal femoral metaphysis (100 slices) defining trabecular bone mass and microarchitecture, including trabecular bone volume fraction (BV/TV; %), trabecular number (Tb.N; 1/mm), trabecular thickness (Tb.Th; mm), trabecular separation (Tb.Sp; mm [higher values are associated with weaker bone]), and the structure model index (SMI), which indicates whether trabeculae are stronger, plate-like (lower values) or weaker, rod-like (higher values). Cortical thickness (Ct.Th; mm) was assessed at the distal femoral metaphysis (50 slices). Micro-finite element analysis (μFEA) was performed at the femoral metaphysis to assess failure load (N; i.e., bone strength) using the manufacture’s software (Scanco Medical AG, Basserdorf, Switzerland; Finite Element-Software Version 1.13). All μCT parameters were derived using the manufacturer’s protocols.

### Statistical analysis

GraphPad Prism v.10.0 was used for statistical analysis; the results were considered to be statistically significant when P ≤ 0.05. For normally distributed data, the differences between two groups were tested for statistical significance using an independent-sample two-tailed t-tests. For data that were normally distributed and when there was more than one group, one-way ANOVA was used, with Tukey’s comparison post hoc test. Where data were not normally distributed, Mann–Whitney U-tests were used to determine statistical significance.

## Data Availability

The RNA-seq and ChIP-seq datasets generated and analyzed during the current study are available in the GEO repository : GSE279410 for he ChIP-sequencing, GSE279411 for the RNA-sequencing
